# Image-based assessment of extracellular mucin-to-tumor area predicts consensus molecular subtypes (CMS) in colorectal cancer

**DOI:** 10.1038/s41379-021-00894-8

**Published:** 2021-09-02

**Authors:** Huu-Giao Nguyen, Oxana Lundström, Annika Blank, Heather Dawson, Alessandro Lugli, Maria Anisimova, Inti Zlobec

**Affiliations:** 1 Institute of Pathology, University of Bern, Bern, Switzerland; 2 Science of Life Laboratory, Department of Biochemistry and Biophysics, Stockholm University, Stockholm, Sweden; 3 Institute of Applied Simulations, School of Life Sciences und Facility Management, Zürich University of Applied Sciences, Wädenswil, Switzerland; 4 Institute of Clinical Pathology, City Hospital Triemli, Zurich, Switzerland; 5 SIB Swiss Institute of Bioinformatics, Quartier Sorge, Lausanne, Switzerland

**Keywords:** Colorectal cancer, Super-resolution microscopy

## Abstract

The backbone of all colorectal cancer classifications including the consensus molecular subtypes (CMS) highlights microsatellite instability (MSI) as a key molecular pathway. Although mucinous histology (generally defined as >50% extracellular mucin-to-tumor area) is a “typical” feature of MSI, it is not limited to this subgroup. Here, we investigate the association of CMS classification and mucin-to-tumor area quantified using a deep learning algorithm, and  the expression of specific mucins in predicting CMS groups and clinical outcome. A weakly supervised segmentation method was developed to quantify extracellular mucin-to-tumor area in H&E images. Performance was compared to two pathologists’ scores, then applied to two cohorts: (1) TCGA (*n* = 871 slides/412 patients) used for mucin-CMS group correlation and (2) Bern (*n* = 775 slides/517 patients) for histopathological correlations and next-generation Tissue Microarray construction. TCGA and CPTAC (*n* = 85 patients) were used to further validate mucin detection and CMS classification by gene and protein expression analysis for *MUC2*, *MUC4*, *MUC5AC* and *MUC5B*. An excellent inter-observer agreement between pathologists’ scores and the algorithm was obtained (ICC = 0.92). In TCGA, mucinous tumors were predominantly CMS1 (25.7%), CMS3 (24.6%) and CMS4 (16.2%). Average mucin in CMS2 was 1.8%, indicating negligible amounts. RNA and protein expression of *MUC2*, *MUC4*, *MUC5AC* and *MUC5B* were low-to-absent in CMS2. MUC5AC protein expression correlated with aggressive tumor features (e.g., distant metastases (*p* = 0.0334), *BRAF* mutation (*p* < 0.0001), mismatch repair-deficiency (*p* < 0.0001), and unfavorable 5-year overall survival (44% versus 65% for positive/negative staining). MUC2 expression showed the opposite trend, correlating with less lymphatic (*p* = 0.0096) and venous vessel invasion (*p* = 0.0023), no impact on survival.

The absence of mucin-expressing tumors in CMS2 provides an important phenotype-genotype correlation. Together with MSI, mucinous histology may help predict CMS classification using only histopathology and should be considered in future image classifiers of molecular subtypes.

## Introduction

Colorectal cancers are a heterogeneous group of tumors, from the histomorphological, clinical and molecular points-of-view. In terms of molecular changes, one particularly well-described genomic aberration is microsatellite instability (MSI), affecting ~15% of all cancers^1^. Patients with sporadic MSI cancers have defects in DNA mismatch repair (MMR) machinery, which is relevant for at least three different scenarios. First, MSI status serves as a diagnostic tool to help identify colorectal cancers arising from a possible familial setting (e.g. Lynch syndrome)^2^. Second, in general, patients with stage II colorectal cancers seem to derive a survival benefit with MSI^3^. Third, evidence suggests that patients with MSI colorectal cancers respond poorly to certain chemotherapies, but may have remarkable positive responses to immunotherapy^1,4^. The MSI status is therefore an important factor affecting treatment decisions in colorectal cancer and is included in both pathological and oncological guidelines (ESMO)^5^.

The MSI status also constitutes the backbone of all major molecular classifications of colorectal cancer today, including the 2015 consensus molecular subtypes (CMS)^6^. The CMS classification summarizes four major subgroups of colorectal cancers mainly from the genetic and epigenetic perspectives, with impact on clinical and therapeutic outcome^7^. CMS1 includes the hypermutated, hypermethylated tumors with frequent *BRAF* mutation and immune cell infiltrates. These are predominantly the MSI cancers. CMS2 include tumors derived from canonical WNT signaling pathway deregulation and have high frequency somatic copy number alterations (37% of all cases). A metabolic subtype with *KRAS* mutations and mixed MSI status is found in CMS3 (13% of cases). Finally, the CMS4 cancers encompass those with mesenchymal and stem-cell like features, and leading to the worst overall and recurrence-free survival.

Attempts have been made to predict MSI cancers using only histopathological features of the cancer. In fact, experienced pathologists can often identify MSI cases simply by glancing at the hematoxylin & eosin (H&E) stained slide. In 2003, work by Jass and colleagues led to the publication of the “MS-Path” score, namely a model for identifying MSI-high, specifically Lynch syndrome patients using a handful of clinical and histopathology features^8^. These included the presence of Crohns’-like reaction, tumor infiltrating lymphocytes and the presence of mucinous histology. Mucin is not exclusively found in MSI-high patients though, and the prognosis associated with mucinous histology in primarily surgically treated colorectal cancer is unclear^9^. Moreover, the WHO classification defines a mucinous tumor as having >50% extracellular mucin to tumor area, which is not only arbitrary but also challenging to report in many cases. Finally, mucin-producing colorectal cancers are themselves heterogeneous and show a wide range of mucin-to-tumor ratio.

In order to better understand the role of extracellular mucin in colorectal cancer, we created a deep learning classifier to quantify the extracellular mucin-to-tumor area ratio in two independent cohorts (Bern *n* = 517, TCGA *n* = 412) and investigate the genotype-phenotype correlation of mucin-to-tumor ratio with CMS groups, MSI status and expression of mucin-producing genes.

## Methods

### Cohorts

#### Cohort 1—Bern

A large retrospective cohort of 517 primary colorectal cancer patients diagnosed at the Institute of Pathology between 2002 and 2018 and treated at the Insel Hospital Bern (Switzerland) were included in this study. All preoperatively treated rectal cancer cases were excluded. Histopathological characteristics were reviewed according to the TNM 7th edition. These included pT and pN classifications, lymphatic, venous and perineural invasion (L, V, and Pn classifications), distant metastasis (clinical or pathologically confirmed at the time of first diagnosis), tumor budding according to the ITBCC criteria^10^, the percentage of the tumor border with expanding growth pattern, the Klintrup–Mäkinen score for peritumoral inflammation and tumor grade. Histological subtype was determined according to WHO (4th ed.) and a mucinous subtype was defined as >50% extracellular mucin per tumor area. MMR-status was determined using immunohistochemistry for the four MMR proteins, as is standard of practice at our institute (MLH1, PMS2, MSH2, and MSH6). Cases were considered MMR-deficient when at least 1 of these proteins was absent. *BRAF* mutational status was obtained after VE1 immunohistochemistry, as previously reported^11^. No distinction between familial and sporadic cases was made. Clinical information included age at diagnosis, tumor size, wherever available and gender.

From each case, 1–2 diagnostic tumor slides with H&E staining were retrieved from the archives of the Institute of Pathology and digitized using a slide scanner (3DHistech). The use of patient data and tissue have previously been approved by the Ethics Committee of the Canton of Bern, Switzerland (KEK2017-01783). All relevant guidelines of the Institute of Pathology, University of Bern, Canton of Bern, Switzerland were followed for the study.

##### Next-generation Tissue Microarray construction (ngTMA^®^)

A previously described next-generation Tissue Microarray using digital pathology for annotation was constructed from these cases^11^, which is a multi-punch tissue microarray containing cores from the tumor center and invasion front.

##### Immunohistochemistry

All ngTMAs were sectioned at 2.5 μm. Stained for MUC2 and MUC5AC was performed by automated staining using a Leica BOND III (Leica Biosystems, Newcastle, UK) immunostainer. Tris buffer (pH 9) at 95 °C for 30 min was used for antigen retrieval (Leica Biosystems). All tissue sections were incubated with the following primary antibodies: MUC2 (catalog no. NCL-MUC2; NCL-MUC-2, clone Ccp58, 1:200 dilution, Leica Microsystems), MUC5AC (catalog no. NCL-MUC5-AC; clone CLH2, 1:200 dilution, Leica Microsystems). Then, all samples were incubated with HRP (Horseradish Peroxidase)-polymer for 15 min and subsequently visualized using 3,3-Diaminobenzidine (DAB) as brown chromogen (Bond polymer refine detection, Leica Biosystems, Ref DS9800) for 10 min.

MUC5AC and MUC2 were scored as positive or negative based on any tumor cell staining or complete absence of staining, respectively. In the case of multiple punches per tissue sample, we collated the results and if positivity was seen in any of the samples, the whole case was considered positive.

#### Cohort 2—TCGA

The Cancer Genome Atlas (TCGA) data^12^ from public repositories at the National Institutes of Health (NIH; USA) was considered in this study. This data source has 1735 H&E whole slide images (WSI), clinical information and genomic data of 553 patients in two projects TCGA-COAD (colorectal adenocarcinoma) and TCGA-READ (rectal adenocarcinoma), available at the GDC data portal (https://portal.gdc.cancer.gov). The clinical information included age at diagnosis, gender, tissue location, tumor size, prior treatment type. Some histopathological characteristics such as pT and pN classifications, and tumor grade were also included. From this resource, we created a sub-dataset, namely TCGA-412 which has 412 patients. Only cases with at least one WSI containing tumor tissues and of good enough quality for analysis were kept. Cases labelled as “preoperative therapy” were removed from the study. Cases with more rare histological subtypes such as neuroendocrine tumors, or signet ring cell tumors were excluded leaving only cases labeled strictly as “mucinous” and “adenocarcinoma”, or non-mucinous. Totally, 871 H&E WSI of TCGA-412 dataset were investigated in this study. All of 412 cases have CMS and MSI labels retrieved from Sage Bionetworks Synapse which were built using a gene expression–based subtyping algorithm^6^. The MSI labels were double-checked with the PreMSIm and MSIseq^13,14^, two MSI prediction R packages based on the expression profiling of a gene panel (exome-sequenced tumors, respectively).

#### Cohort 3—CPTAC

A public dataset, namely The Clinical Proteomic Tumor Analysis Consortium (CPTAC) from the National Cancer Institute (NCI; USA) was also included. The 373 H&E WSIs of 106 colon adenocarcinoma patients were downloaded from The Cancer Imaging Archive (TCIA)^6^. The clinical, proteomic and genomic data are available at LinkedOmics (http://linkedomics.org/cptac-colon/). Clinical information included age at diagnosis, gender, tumor site, vital status and some histopathological characteristics such as pT and pN classifications, tumor stage, the presence of vascular, lymphatic, perineural invasion or colon polyps and the mutation status of *POLE, KRAS, NRAS, BRAF, MLH1, MSH2, PMS2, MSH6*. The MSI status was detected by fluorescent PCR-based assay^15^. There were 85 patients assigned into four CMS groups using a classifier from Sage Bionetworks Synapse based on the RSEM gene expression profiles from RNA-Seq^6^. A sub-dataset with 231 H&E WSIs of these 85 patients with CMS information was available. However, these images correspond to frozen sections, rather than H&E from formalin-fixed paraffin embedded material and we refrained from including the images into this study.

### Weakly supervised tissues segmentation from prior information in CRC

**Fig. 1:**
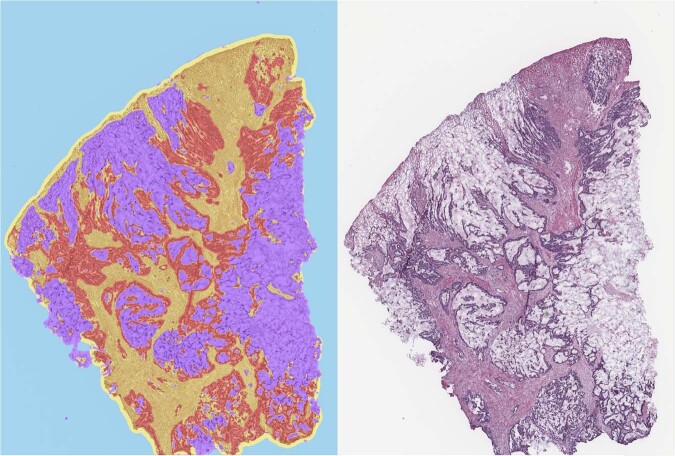
Example of tissue detection in H&E CRC slides using the GAWS algorithm, with mucin in purple, tumor in red, normal tissues in yellow, background and artifact in cyan. The number of tissue classes can be modified by changing the number of cluster parameter of the proposed algorithm.

Here we propose a novel precise tissue segmentation of histopathology images using deep learning, namely by Group Affinity Weakly Supervised segmentation (GAWS). It processes one histopathology image and some patches of prior tissue as input with three main steps. First, an output image is created during a forward process by extracting the visual feature of each pixel from a convolutional neural network and assigning it into different clusters. Then, a target image is created by refining the output image with the similarity constraints on prior tissue pattern, color, and spatial distribution of pixels. Finally, a backpropagation process based on a segmentation loss function evaluates the error signals between output and target images, and updates the network parameters. Supplementary Fig. 1 shows the main schema of the proposed GAWS method. An example result of final output of the proposed algorithm with the WSI is shown in Fig. 1.

Two cohorts are included in this study. Tissues vary significantly in morphology, scale, texture and color distribution, which makes it difficult to find a general pattern for each tissue type. Thus, a weakly supervised segmentation is particularly well suited for these problems, i.e., we do not know the optimal number of tissue types in the image. Prior tissue is needed to guide the creation of a target image by forcing all pixels related to this tissue into a same cluster label and guide the algorithm to select the best candidates for segmentation. In practice, there are two ways to perform the prior tissue selection. An expert pathologist manually selects a few example patches of tissues of interest by using some stand-alone software, such as QuPath^16^ and ASAP (https://github.com/computationalpathologygroup/ASAP). On the other hand, existing automatic extraction tools using deep learning to classify and locate the tissue of interest can be applied^17–19^. Especially in our previous work^19^, we proposed a system for tissue detection in WSI based on an ensemble learning method with two raters, a VGG^20^ and a CapsuleNet^21^. Some additional examples of the tissue segmentation output are shown in Supplementary Fig. 2.

### Statistical analysis

Descriptive statistics for all variables were carried out. To determine the association between categorical variables, the Chi-square test was used. For analysis of two continuous variables, the Spearman’s correlation coefficient was performed and to evaluate the relationship between a quantitative variable among different categories of another variable, the Wilcoxon’s Rank Sum or Kruskal–Wallis test was performed. Receiver Operating Characteristics (ROC) curve analysis was performed to determine the most discriminating cut-off for MMR-deficiency in the Bern cohort, determined to be 10%. Intra-observer agreement for % mucin values between pathologists’ scores and algorithm was analyzed using the Intraclass Correlation Coefficient (ICC), with values >0.8 considered excellent. Survival analysis was carried out using log-rank statistics and Kaplan–Meier curves as well as Hazard Ratios and 95% CI from Cox regression analysis. *P* values were two-sided and considered significant when *p* < 0.05.

## Results

### Agreement between deep learning algorithm for mucin-to-tumor area quantification and pathologists’ scores (Bern cohort)

First, we tested the inter-observer agreement of extracellular mucin component, recorded as the percentage of total tumor area covered by extracellular mucin, between two pathologists. One hundred and forty-nine H&E scans from the Bern cohort were used for this purpose. The ICC was 0.92, indicating excellent agreement. We next tested the unsupervised GAWS algorithm against the scores from both pathologists. Again, results were excellent, with pathologist 1 ICC = 0.915 (95% CI: 0.885–0.937) and pathologist 2 ICC = 0.923 (95% CI: 0.896–0.943). Based on these results, we confidently applied the algorithm to the remaining 775 H&E slides from the Bern cohort, and 871 slides from the TCGA cohort. In the case where more than one slide/case was available, the largest value across all slides was used for further analysis.

### Association of extracellular mucin, CMS and outcome in the TCGA cohort (Table 1)

In the TCGA cohort, an association between TNM stage and CMS was observed. CMS2 and CMS4 tumors were more frequently stage IV (17.8 and 18.2%) compared to CMS1 (4.4%) and CMS3 (3.4%), while stage I tumors were most often CMS3 (*p* < 0.0001). The interconnectivity between CMS and stage has recently also been reported in this cohort^22^. MSI-H was observed in CMS1 (81.4%) and CMS3 (19.7%), and rarely in CMS2 and CMS4 (1.2% and 5.4%, respectively) (*p* < 0.0001). Similarly, tumors with mucinous histological subtype were observed in CMS1 (25.7%) and CMS3 (24.6%) and less frequently in CMS4 (16.2%). Of the three cases declared as mucinous in CMS2, our algorithm detects no mucin, which was confirmed by evaluating the image by pathologist. These tumors are likely mislabeled, signifying that CMS2 tumors exclude those with mucinous subtype. These results are again supported by evaluating the percentage of extracellular mucin/tumor area, showing similar mean values for CMS1 and CMS3 (20.9 and 20.4%), followed by CMS4 (18.8%) and low-to-no mucin in CMS2 (1.8%).

Although overall, no significant difference was noted between non-mucinous and mucinous cancers, pronounced differences were observed in stage II and CMS3 subgroups. In stage II cancers, mucinous histology leads to poor 5-year OS (non-mucinous vs mucinous cancer 94.5% and 76.5% respectively, *p* = 0.0276) (Supplementary Fig. 3A, B). Similarly, in CMS3 patients with mucinous cancers do significantly worse than patients with non-mucinous cancers (58.5% versus 95.2% 5-year OS; *p* = 0.0052). However, in both cases, the impact of mucinous histology was not independent of postoperative therapy information, when adjusting confounding. Details can be found in Supplementary Table 1.

### Subgroup analysis of MSI and MSS tumors by mucinous histology and CMS groups

**Fig. 2:**
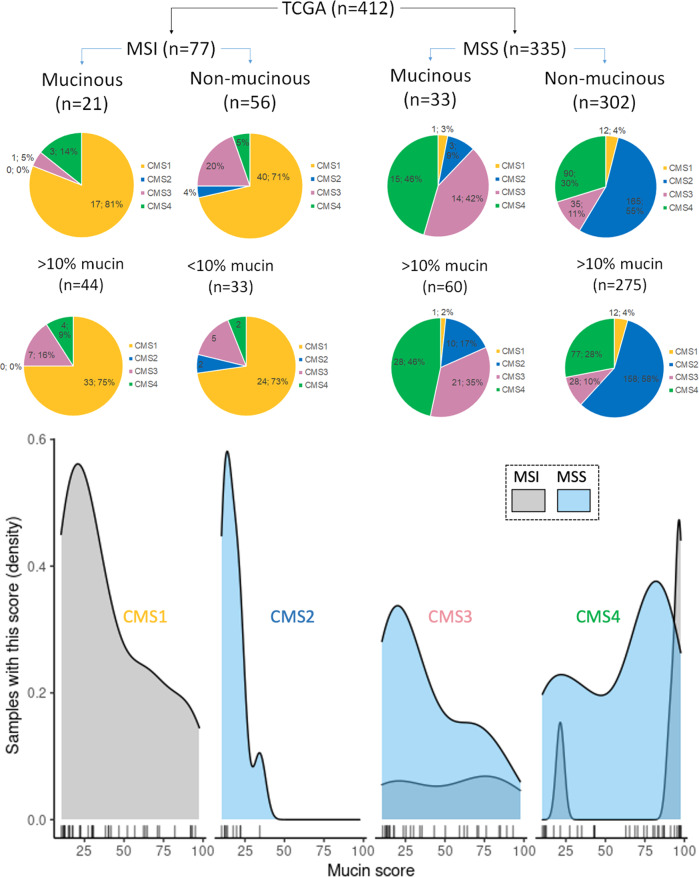
Subgroup analysis of MSI/MSS tumors by mucinous histology and CMS groups. (Pie-Chart) Distribution of CMS classifications by MSI status in (first row) mucinous and non-mucinous (adenocarcinoma) colorectal cancers and (second row) high or low mucin content based on 10% threshold from the TCGA database. (Line-Chart) Plot of the density of each mucin score by MSI status across each CMS group in mucinous samples (mucin score >10%). The x-axis shows the %-mucin detected by the AI algorithm with each line representing a sample with that value. In CMS1 and CMS3, mucin values are distributed throughout the range of possible values from 0–100%, while no case in CMS2 reaches the threshold of 50% to be declared as “mucinous”. CMS4 points towards a bimodal distribution of mucin scores.

MSI-H tumors are not exclusive to the CMS1 subgroup. They occur albeit with less frequency in CMS3 and only rarely in CMS2 and CMS4. Mucinous histology seems to be independent of MSI status (Fig. 2). We looked at the data in the TCGA cohort two ways. In the first approach using the TCGA cohort, 21 mucinous cancers were MSI compared to 33 MSS. Observing the results, we then used the ROC-derived threshold value of 10% to classify tumors as low/high mucin. 57% of MSI tumors had >10% extracellular mucin in contrast to 27% which are classified as “mucinous” cancers. In the MSS, 17.9% have >10% extracellular mucin and 9.9% (*n* = 33) are classified as having mucinous histology. Of these, 15 are CMS4 and 14 are CMS3 tumors. Figure 2 also shows the exact values of extracellular mucin as a function of the number of patients (density) with each value. Here, results clearly support that the distribution of mucin throughout CMS1 and CMS3 covers all ranges of values, that CMS2 is a low-to-no mucin-producing group and finally that CMS4 shows a bimodal distribution of mucin values.

#### Association of expression of mucin-associated genes and CMS groups in TCGA

**Fig. 3:**
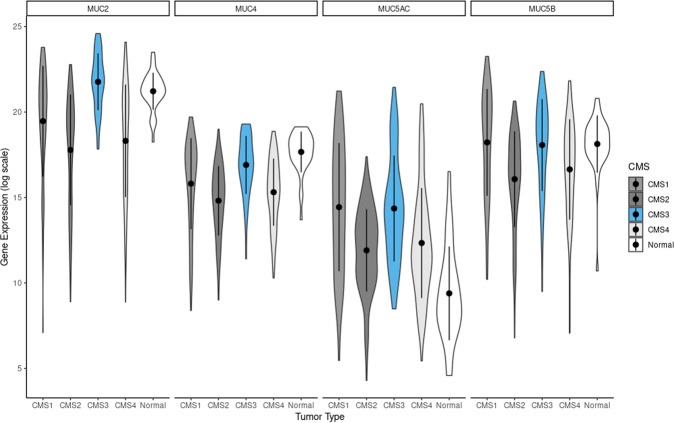
Violin plots of mucin-related gene expression data from the TCGA-COAD and TCGA-READ datasets. Values on the y-axis represent log scale of the normalized mRNA sequencing counts. On the x-axis, different CMS types of primary tumors are shown along with normal tissue sample values.

In order to validate the association of mucin detected by our deep learning classifier and CMS groups, we used a second complementary approach by analyzing the TCGA and CPTAC cohorts for gene expression and protein expression namely for four well-known mucin-related genes: *MUC2, MUC4, MUC5AC* and *MUC5B*. The expression of mRNA (TCGA) of all the four mucin-related genes is at significantly lower levels or nearly absent in CMS2 (Fig. 3), in line with the results from the evaluation of our images. For protein expression (CPTAC), obtained through tandem mass tag labeling, the differences were less pronounced for MUC5AC and MUC5B, where a handful of samples in the CMS2 group had high expression levels (See Supplementary Fig. 4 for CPTAC data).

### Clinical impact of mucin and mucin-related proteins in the Bern cohort

#### Associations of mucinous histology and extracellular mucin with clinicopathological features

Mucinous histological subtype, as taken from diagnostic reports, was only associated with a less advanced pT category. The average percentage of mucin in these cases was 63%. Evaluating the result of the AI algorithm shows that a higher amount of extracellular mucin is related to right-sided tumor location (*p* = 0.0026), more advanced pT stage (*p* = 0.0485), higher tumor grade (*p* < 0.0001), but less venous invasion (*p* = 0.0039) and a higher percentage of expanding tumor border (*p* = 0.0001). The mean percentage of mucin in MMR-deficient tumors was 27.1% compared to 8.4% in MMR-proficient cases (*p* = 0.0001). Using a 10% cutoff to declare a case as “high” mucin, the above associations were maintained. In addition, a higher percentage of mucin correlated with lower tumor budding (*p* = 0.0241), higher Klintrup–Mäkinen score (*p* = 0.0577) and *BRAF* mutation (*p* = 0.0329).

#### Clinical impact of MUC5AC and MUC2 in colorectal cancer

**Fig. 4:**
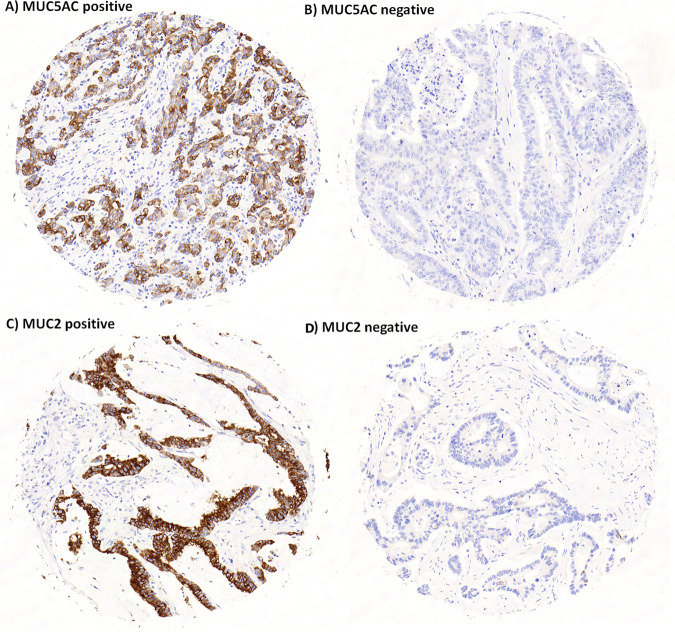
Representative immunohistochemistry images for MUC5AC and MUC2 in colorectal cancer. MUC5AC positive (**A**) and negative (**B**), MUC2 positive (**C**) and negative (**D**).

**Fig. 5:**
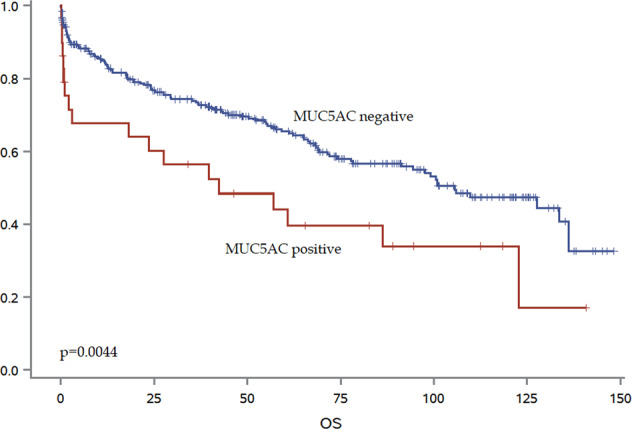
Kaplan-Meier curve showing survival time differences with MUC5AC expression.

MUC5AC and MUC2 were analyzed on an ngTMA of 337 patients (Fig. 4). Expression of both proteins was associated with mucinous histology (*p* < 0.001) (Table 2). MUC5AC was additionally associated with right-sided tumor location (*p* = 0.01), higher pT (*p* = 0.0073), more frequent distant metastasis (*p* = 0.0334), higher tumor grade (*p* < 0.0001), frequent *BRAF* mutation (*p* < 0.0001) and MMR-deficiency (*p* < 0.0001). Positive expression was an unfavorable prognostic factor (5-year OS, 44% versus 65% for positive and negative staining) (Fig. 5). MUC2 expression correlated with higher tumor grade (*p* = 0.0044), a more expansive border (*p* = 0.044) but with less lymphatic and venous invasion (*p* = 0.0096, *p* = 0.0023, respectively). A trend toward more frequent MSI was found (*p* = 0.0567).

**Table 2:** Association of MUC5AC and MUC2 expression in colorectal cancer using a multiple punch ngTMA (Bern cohort, total 373 patients).

		MUC5AC			MUC2		
Feature		Negative (*n* = 306)	Positive (*n* = 29)	*P* value	Negative (*n* = 221)	Positive (*n* = 116)	*P* value
Age (years)	Mean	69.4	70.4	0.797	69.8	68.9	0.6564
Gender	Female	117 (38.0)	16 (55.2)	0.0703	86 (38.9)	47 (40.5)	0.7748
	Male	191 (62.0)	13 (44.8)		135 (61.1)	69 (59.5)	
Histological subtype	Non-mucinous	283 (92.5)	21 (72.4)	**0.0004**	210 (95.0)	94 (82.5)	**0.0002**
	Mucinous	23 (7.5)	8 (27.6)		11 (5.0)	20 (17.5)	
Tumor location	Left	137 (46.3)	3 (12.5)	**0.001**	99 (46.7)	41 (38.0)	0.154
	Rectum	57 (19.3)	4 (16.7)		42 (19.8)	19 (17.6)	
	Right	102 (34.5)	17 (70.8)		71 (33.5)	48 (44.4)	
pT	pT1	4 (1.3)	2 (6.9)	**0.0073**	1 (0.5)	5 (4.4)	0.0813
	pT2	51 (16.8)	2 (6.9)		34 (15.5)	19 (16.7)	
	pT3	168 (55.3)	11 (37.9)		120 (54.8)	59 (51.8)	
	pT4	81 (26.6)	14 (48.3)		64 (29.2)	31 (27.2)	
pN	pN0	134 (44.2)	11 (40.7)	0.0652	89 (40.8)	56 (50.0)	0.2773
	pN1	102 (33.7)	5 (18.5)		74 (33.9)	33 (29.5)	
	pN2	67 (22.1)	11 (40.7)		55 (25.2)	23 (20.5)	
pM or CM	Absent	227 (73.7)	16 (55.2)	**0.0334**	10 (72.4)	83 (71.6)	0.8692
	Present	81 (26.3)	13 (44.8)		61 (27.6)	33 (28.4)	
Tumor grade	G1	8 (2.6)	0 (0.0)	**<0.0001**	3 (1.4)	5 (4.3)	**0.0044**
	G2	239 (77.9)	13 (44.3)		177 (80.5)	75 (64.7)	
	G3	60 (19.5)	16 (55.2)		40 (18.2)	36 (31.0)	
Lymphatic	L0	86 (30.6)	7 (30.4)	0.9864	50 (25.5)	43 (39.8)	**0.0096**
	L1	195 (69.4)	16 (69.6)		146 (74.5)	65 (60.2)	
Venous	V0	125 (44.2)	12 (52.2)	0.4578	76 (38.4)	61 (56.5)	**0.0023**
	V1	158 (55.8)	11 (47.8)		122 (61.6)	47 (43.5)	
Perineural	Pn0	223 (80.8)	16 (69.6)	0.1963	148 (76.7)	91 (85.8)	0.0584
	Pn1	53 (19.2)	7 (30.4)		45 (23.3)	15 (14.2)	
Klintrup–Mäkinen	KM0	23 (8.4)	3 (13.0)	0.1995	19 (9.9)	7 (6.6)	0.2317
	KM1	118 (42.9)	9 (39.1)		81 (42.2)	46 (43.4)	
	KM2	108 (39.3)	6 (26.1)		68 (35.4)	46 (43.4)	
	KM3	26 (9.5)	5 (21.7)		24 (12.5)	7 (6.6)	
Tumor border % expansive	Mean	44.6	49.5	0.5114	42.1	50.2	**0.044**
Nr. ITBCC buds	Mean	9.3	10.7	0.3057	9.7	9.0	0.2691
Post-op therapy	Absent	234 (76.0)	20 (69.0)	0.4023	165 (74.7)	89 (76.7)	0.6761
	Present	74 (24.0)	9 (31.0)		56 (25.3)	27 (23.3)	
BRAF VE1	Wild-type	187 (92.1)	2 (12.5)	**<0.0001**	132 (88.0)	57 (82.6)	0.2811
	Mutation	16 (7.9)	14 (87.5)		18 (12.0)	12 (17.4)	
MSI	Deficient	16 (9.2)	11 (68.8)	**<0.0001**	14 (10.9)	13 (21.3)	0.0567
	Proficient	157 (90.8)	5 (31.2)		114 (89.1)	48 (78.7)	
OS	5-years %	65.4	44.0	**0.0039**	63.6	63.4	0.9707

Bold values indicate statistical significance *p* < 0.05.

## Discussion

In this study, we combine histomorphology with deep learning and genomics to validate the relationship of mucin-producing colorectal cancers and CMS classification. The novel findings of this study suggest that CMS2 cancers generally lack mucin, which is reflected not only in the quantification of extracellular mucin to-tumor area resulting from our algorithm, but also in analysis of mucin-related genes and proteins in two different publicly available datasets. We additionally show that although mucin is a feature of MSI cancers, it is by far not limited to the subgroup. Moreover, the specific MUC5AC protein expression is associated with aggressive cancers and worse overall survival (Fig. 5).

In a first step, we developed a novel deep learning algorithm using a segmentation algorithm to help quantify the extracellular mucin across two large cohorts (Bern and TCGA) and takes advantage of weakly supervised learning from a predefined tissue pattern without any pre-trained parameters of the neural network or training data. The proposed method can be considered as a potential visual scoring tool with an efficiency in processing time and memory needed to support pathologists and to overcome the practical limitations of visual scoring. Since the inter-observer agreement between pathologists and the proposed algorithm was excellent (ICC = 0.917), we applied it across all of our images, totaling >1500 slides.

In a second step, we evaluated the association of extracellular mucin/tumor area and the CMS classification on the TCGA dataset. Our results clearly show that (1) CMS2 tumors lack mucin, (2) mucin-producing tumors can be CMS1, CMS3 and CMS4, (3) mucin-producing tumors may or may not be MSI and (4) CMS4 cancers can be separated into low- or high-mucin-producing cancers.

Our results indicate that extracellular mucin quantified on the H&E slide by histology can be an indication of the CMS group, especially for cancers that are MSS. Such genotype-phenotype correlations have already been described, albeit without the use of digital pathology. Jenkins and colleagues in 2005, developed the MS-Path score using a multivariable logistic regression model for the prediction of Lynch syndrome patients, based solely on two clinical (age and tumor location) and four histopathological features (histological subtype including mucinous, tumor grade, Crohns’-like reaction and tumor infiltrating lymphocytes), reaching an AUC value of >0.8^8^. In fact, well-trained gastrointestinal pathologists can, with high accuracy, predict the occurrence of MSI-high colorectal cancers simply from the impression of these features in an H&E slide. In a second publication, Jass and coworkers found that mucinous differentiation of colorectal cancers was significantly greater in tumors with *KRAS* mutations, which is also a frequent feature of CMS3^6,23^. Jang and colleagues apply deep learning to images from the TCGA cohort with the aim of differentiating between non-mutated and *KRAS* mutated colorectal cancers^24^. They extract information on *APC, KRAS, PIK3CA, SMAD4*, and *TP53* mutations and show a modest degree of accuracy in terms of classification. Echle and colleagues performed MSI detection from 8836 colorectal cancers of mixed stages from multiple institutions in Europe, UK, and USA^25^. They impressively achieve clinical grade performance with an AUC of 0.92 in the development cohort and 0.95 in the validation cohort although the histological features leading to the classification are not discussed. Extending the idea of genotype-phenotype correlation across many tumor types, Noorbakhsh et al. observe similar histological patterns between TP53 mutated cancers independent of tumor entity, and report whole slide image- and cross-tissue AUC values of 0.65–0.8^26^.

In this study, we go one step further to analyze two different publicly available datasets with genomic or proteomic data and CMS classification. We analyzed four common mucin-related genes, namely *MUC2, MUC4, MUC5AC* and *MUC5B*. Both analyses on the gene expression (TCGA) and protein expression (CPTAC) level clearly show that CMS2 tumors demonstrate low expression of all genes or proteins, followed by CMS4 tumors, explained by the bimodal distribution of mucin within this particular CMS. We also note a high expression of MUC5AC protein (CPTAC) in CMS1, an entirely MSI-H group and high MUC2 expression in CMS3. In fact, our results indicate that MUC2 expression may play a role in differentiating CMS3 and CMS4 cancers. These results are again reflected in our ngTMA immunohistochemistry analysis, showing a strong association between MMR-deficiency and MUC5AC expression. Interestingly, although MUC5AC positive cancers are associated with significantly worse overall survival in patients, MUC2 cancers show a much more indolent phenotype. This is in line with in vitro studies showing that MUC5AC expression enhanced cell invasion and migration, decreased apoptosis and led to tumorigenesis and appearance of metastatic lesions in orthotopic mouse models^27^. In addition, MUC5AC leads to resistance to 5-FU based chemotherapy, reflecting the situation of patients in CMS1 with predominantly MSI-H cancers^6^. Moreover, a systematic review including MUC2 indicates an improved outcome with MUC2 over-expression in colorectal cancer patients^28^ again, in line with our findings.

In fact, several additional features from the histology seem to be closely related to the CMS classifications. Sirinukunwattana et al. apply deep learning methods to H&E images of colorectal cancer cohorts with known CMS analysis^29^. They achieve excellent AUC values using this image CMS (imCMS) classifier and highlight common histopathology features that seem to occur in each group. For example, they show that mucinous differentiation and lymphocytic infiltration are common in imCMS1, whereas a prominent desmoplastic stroma occurs in imCMS4. They note cribriform growth patterns and comedo-like necrosis in imCMS2 while imCMS3 is characterized by mucin-filled glandular structures. Our previous work on tumor budding in several cohorts with CMS classification also identifies high-grade budding as a feature closely related to CMS4^30^.

Finally, although the focus of this study was not on the diagnostic reporting of mucinous histology, some observations can be made. “Mucinous” cancers in both cohorts are not always in line with the WHO definition of >50% extracellular mucin, especially in the TCGA cohort, where the median extracellular value is 50% (indicating that half the so-called mucinous cases are below that value). One possibility is that the images included for the dataset are not the most representative of that cancer. Our mucin-detection algorithm also found discrepant cases, which were confirmed by an expert pathologist. It also suggests that pathologists’ impression of what constitutes a mucinous cancer relies also on additional histological impressions, not only on mucin content.

To summarize, extracellular mucin detected by deep learning from an H&E image may help to differentiate between CMS groups, with CMS2 tumors generally lacking mucin, independently of MSI status. Together with MSI, mucinous histology may help predict CMS using only histopathology and should be considered in future image classifiers of molecular subtypes.

## Supplementary information

Supplemental figures and tables

## Data Availability

TCGA and CPTAC datasets are publicly available datasets. Bern cohort data is not currently publicly available, but are available from the corresponding author on reasonable request.
