# Supplementary material for: Image-based assessment of extracellular mucin-to-tumor area predicts consensus molecular subtypes (CMS) in colorectal cancer
Source: Mod Pathol. 2021 Sep 2;35(2):240–8. doi: 10.1038/s41379-021-00894-8 (PMC8786661; doi:10.1038/s41379-021-00894-8)
Supplement: Supplementary file 1 — Supplemental figures and tables [file 41379_2021_894_MOESM1_ESM.docx]

**Supplemental figures and tables**

Figure 1: Main schema of our proposed approach, using an H&E stained image, which can be divided into three main steps. Step1: visual feature extraction and classification to create the output image. A feature map tensor is created by some convolutional components, where each component has a convolutional layer, a group normalization and a ReLU activation function.; Step2: spatial and color refinement (superpixel cluster) and prior tissue matching to create the target image; Step3: backpropagation process to update the network. The loss between the network responses (Output) and the refined cluster labels (Target) guides the backpropagation to update the parameters of the convolutional filters and classification layer.


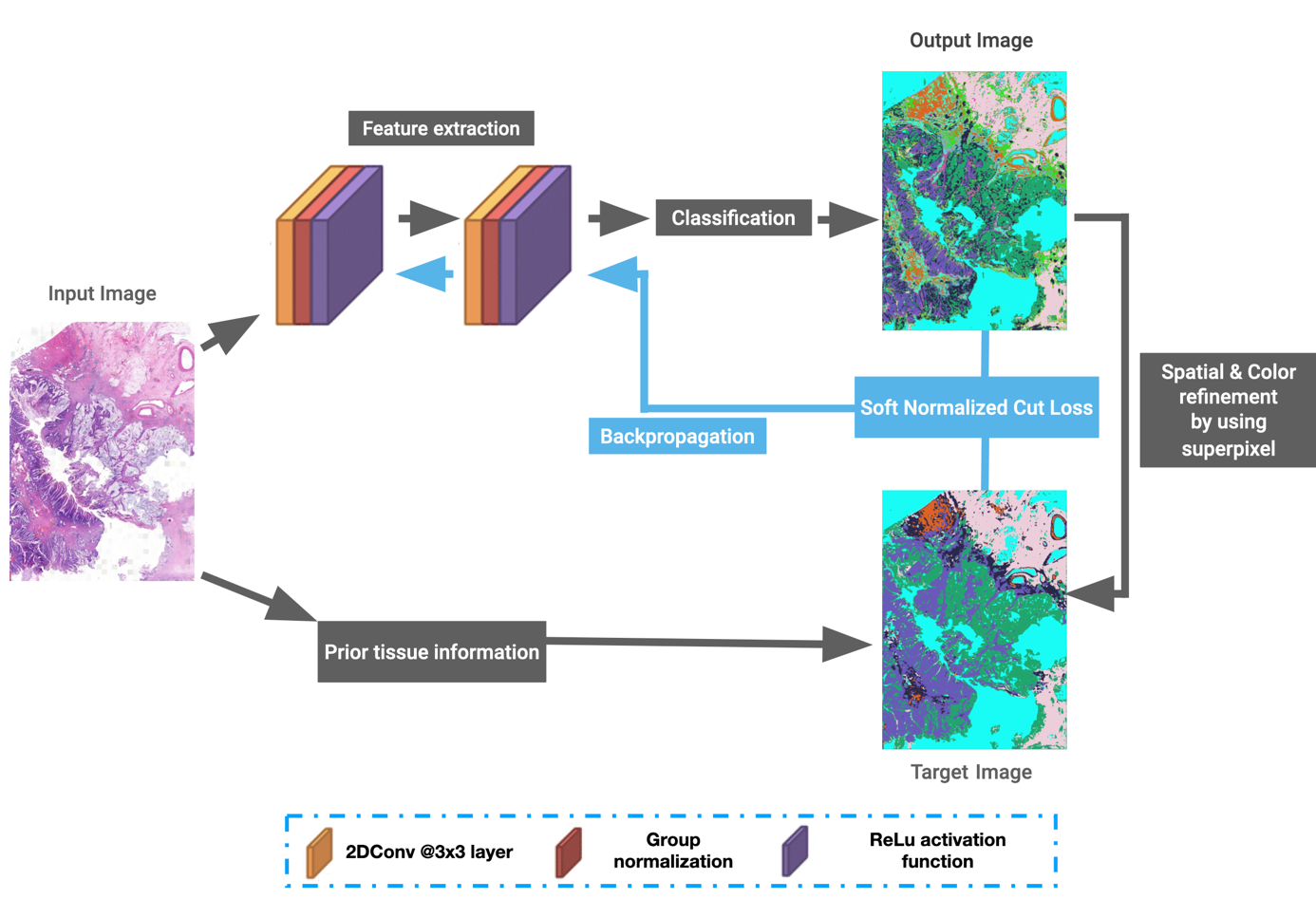


Figure 2: Some other result examples of the proposed tissue segmentation algorithm for all of three cohorts: Bern (first row), TCGA (second row) and CPTAC (last row). The original image is in the left column. The overlay tissues segmentation is in the right column, where mucin in purple, tumor in red, normal epithelium in green, other normal tissues in yellow, background & artifact in cyan.


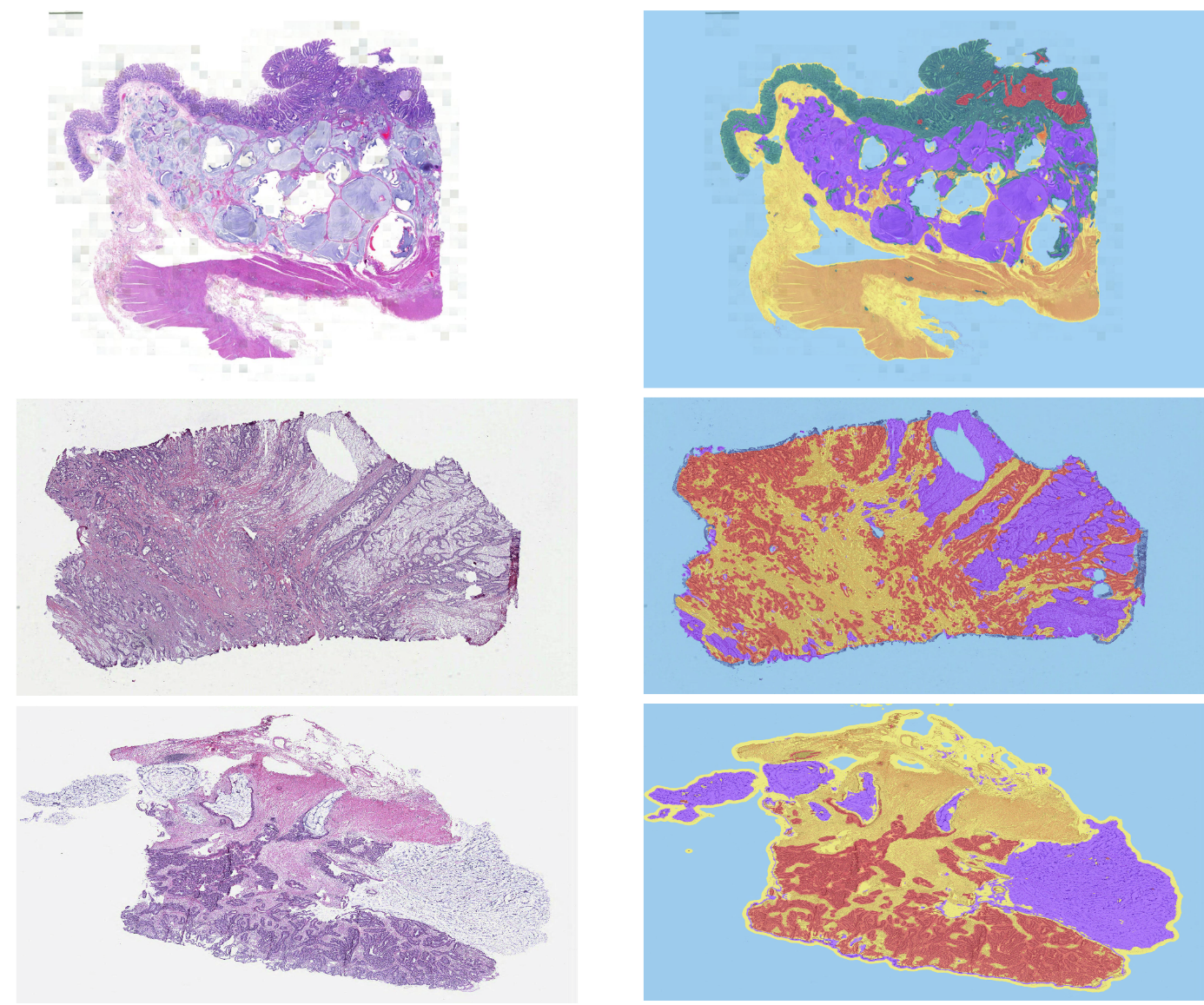


Figure 3: Kaplan-Meier curves showing the impact of mucinous or non-mucinous differentiation on overall survival (OS) in the TCGA database, in stage II patients and in CMS3. In both cases, mucinous histology is significantly associated with worse OS.


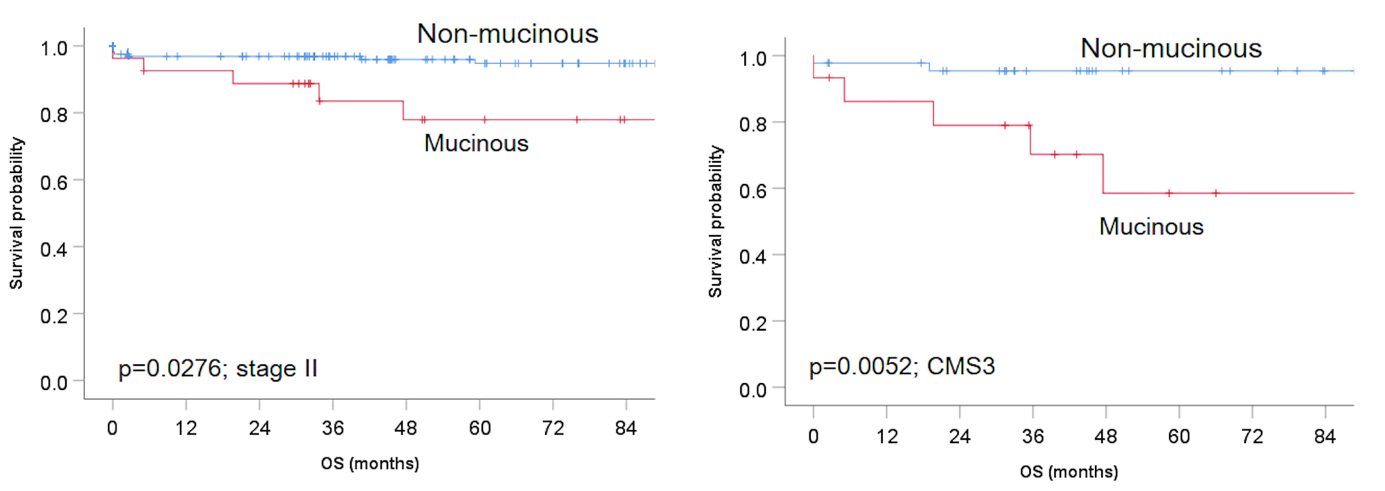


Figure 4: Violin plots of mucin-related proteins expression data from CPTAC-COAD

dataset tumor samples. Values on the y-axis represent log-ratio normalized tandem mass tag TMT) values. On the x-axis different CMS types of tumors are shown along with normal tissue values. Samples labelled as “Unknown CMS” are not shown on the plot.


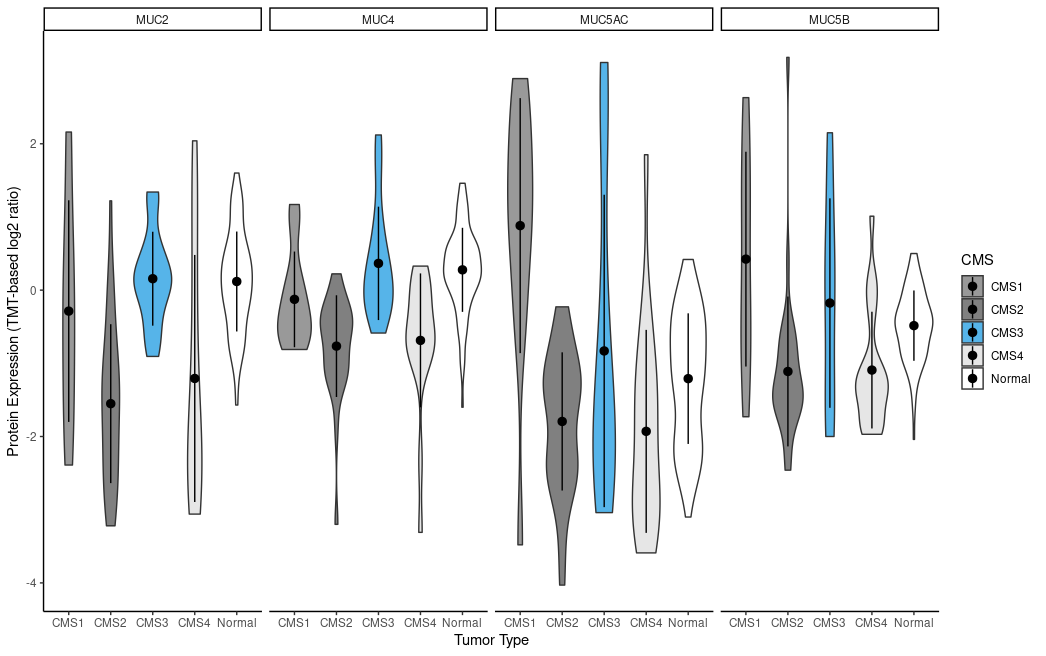


**Table 1:** **Overall survival time differences between varying TNM stages and CMS groups by histological subtype**

|  |  | 5-year OS% | No. total | No. events | Log-rank p-value |
| --- | --- | --- | --- | --- | --- |
| Histology | Non-mucinous | 88.7 | 400 | 52 | 0.4079 |
|  | Mucinous | 80.9 | 59 | 10 |  |
|  |  |  |  |  |  |
| Stage I | Non-mucinous | 98.9 | 77 | 2 | 0.718 |
|  | Mucinous | 100 | 11 | 0 |  |
|  |  |  |  |  |  |
| Stage II | Non-mucinous | 94.5 | 157 | 12 | **0.0276** |
|  | Mucinous | 76.5 | 25 | 6 |  |
|  |  |  |  |  |  |
| Stage III | Non-mucinous | 85.5 | 105 | 18 | 0.4826 |
|  | Mucinous | 76.2 | 19 | 4 |  |
|  |  |  |  |  |  |
| Stage IV | Non-mucinous | 68.7 | 49 | 16 | 0.2356 |
|  | Mucinous | 100 | 4 | 0 |  |
|  |  |  |  |  |  |
| CMS1 | Non-mucinous | 84.7 | 46 | 7 | 0.6035 |
|  | Mucinous | 94.4 | 18 | 2 |  |
|  |  |  |  |  |  |
| CMS2 | Non-mucinous | 87.4 | 153 | 21 | 0.5095 |
|  | Mucinous | 50 | 3 | 1 |  |
|  |  |  |  |  |  |
| CMS3 | Non-mucinous | 95.2 | 43 | 4 | **0.0052** |
|  | Mucinous | 58.5 | 15 | 5 |  |
|  |  |  |  |  |  |
| CMS4 | Non-mucinous | 85.9 | 85 | 13 | 0.7978 |
|  | Mucinous | 83.6 | 14 | 2 |  |
